# Clinical and polysomnographic characteristics in adults referred to the sleep laboratory: a single-center study

**DOI:** 10.5339/qmj.2022.14

**Published:** 2022-03-02

**Authors:** Samrad Mehrabi, Soroush Bagheri

**Affiliations:** ^1^Division of Pulmonology, Department of Internal Medicine, Shiraz University of Medical Sciences, Shiraz, Iran E-mail: mehrabis@sums.ac.ir; ^2^Department of Internal Medicine, Shiraz University of Medical Sciences, Shiraz, Iran

**Keywords:** sleep apnea syndrome, risk factors, polysomnography, snoring, comorbidity

## Abstract

Background: Polysomnography is the gold standard diagnostic method for obstructive sleep apnea syndrome, and on-time treatment can help prevent further complications of obstructive sleep apnea. However, polysomnography is associated with some difficulties for the patients and physicians, which hinder its application. This study aimed to evaluate the clinical features and polysomnography findings of patients with obstructive sleep apnea.

Methods: Data were retrospectively collected from polysomnography studies at the Sleep Laboratory of Namazi Hospital, Shiraz, Iran, from February 2013 to December 2017. Polysomnography was performed for any patients suspected of obstructive sleep apnea. The researcher reviewed the data extracted and selected the essential clinical features for the statistical analysis. The association of variables with the polysomnography findings was analyzed.

Results: Significant associations were observed between the following factors and severity of obstructive sleep apnea: older age (p = 0.01), snoring (p = 0.122), history of sleep disorders (p = 0.11), no sedatives before sleep (p = 0.039), nocturia (p = 0.001), apnea (p = 0.035), no smoking (p = 0.039), no substance abuse (p = 0.011), hypertension (p = 0.001), cardiac diseases (p = 0.025), and overweight and obesity (p < 0.001).

Conclusion: Considering the concomitant occurrence of obstructive sleep apnea with obesity, hypertension, cardiac disease, snoring, and observed apnea, polysomnography is recommended in these patients before further assessments.

## Introduction

Obstructive sleep apnea syndrome (OSAS), also known as obstructive sleep apnea–hypopnea syndrome, is a respiratory sleep disorder that causes the cessation of breathing and airflow during sleep because of recurrent upper airway collapse.^1^ Its prevalence is approximately 22% in men and 17% in women.^2^ Several risk factors have been suggested, including obesity, male gender, family history of obstructive sleep apnea, large neck circumference, allergic rhinitis or other causes of nasal congestion, adenotonsillar hypertrophy, smoking, alcohol consumption before sleep, and systemic diseases, such as asthma, hypertension, diabetes mellitus, hypothyroidism, and acromegaly.^3^

Obstructive sleep apnea causes sleep problems, excessive daytime sleepiness, and impaired daytime function. If left undiagnosed and untreated, it is also associated with several clinical conditions, such as metabolic dysfunction (such as diabetes mellitus)^4^, accelerated atherosclerosis,^5^ coronary events, cardiovascular death,^6,7^ and psychological diseases such as depression.^8,9^ Therefore, an appropriate diagnosis of obstructive sleep apnea is essential, for which polysomnography (PSG) is considered the gold standard diagnostic method.^10^

During the sleep study by PSG, brain and muscle activities, cardiac rhythm, and oxygen saturation are monitored, which enable PSG not only to detect sleep problems, such as latency of sleep or rapid eye movement (REM) sleep, number of awakenings, sleep duration, and duration of each stage of sleep, but also medical problems, such as respiratory and cardiac dysfunctions.^11^ Despite these advantages, PSG is not an easy test, as the patient has to spend at least one night at the laboratory. Moreover, home-based portable monitoring may not be as accurate as laboratory-based PSG in all patients.^12^ Furthermore, PSG is unavailable at many centers and is an expensive test.^13^ Thus, many patients neglect to undergo this test, in whom the diagnosis of obstructive sleep apnea may be missed and may be affected directly by the main problem and/or indirectly by obstructive sleep apnea complications.

Considering the high prevalence of obstructive sleep apnea in the general population,^2^ investigating the clinical factors associated with obstructive sleep apnea is crucial to determine the probability of obstructive sleep apnea before PSG and perform PSG for high risk cases.

## Materials And Methods

### Study design

This retrospective study was performed at the Sleep Laboratory of Namazi Hospital, Shiraz. Data of patients who underwent PSG from February 2013 to December 2017 at this center were retrospectively collected from the PSG apparatus and paper charts from patients’ medical records. To maintain privacy, data extraction and analysis were performed with patients’ codes rather than with names. The ethics committee of Shiraz University of Medical Sciences approved the study protocol (code: IR.sums.med.rec.1397.247).

Patients had one or some of the following symptoms: excessive daytime sleepiness, snoring, observed apnea, choking and awakening during sleep, and showing disinterest, suspected of obstructive sleep apnea, and PSG was performed at our center. All patients who underwent PSG at our center during the study period were included following a consensus method. Data extracted included demographic characteristics, sleep problems, sleep pattern, sleep habits, breathing status during sleep, restlessness, daytime sleepiness, substance abuse, social status, occupational status, medical history, drug history, underlying diseases, and history of assessing sleep disorders. Of these factors, the following were selected by the researcher and recorded in the study checklist for this study: age, sex, weight, height, body mass index (BMI), history of sleep problems, snoring, history of using sedatives before sleep, number of times waking for urination during sleep, nightmare, apnea witnessed by others, history of waking with a feeling of choking or breathing problems, restless leg or arm during sleep, taking a nap or feeling sleepy during the day, substance abuse, and histories of underlying diseases such as cardiac disease, pulmonary disease, stroke, diabetes, depression, hypertension, and gastroesophageal reflux disease. BMI was calculated by dividing the participant's weight in kilograms to squared height in meters, and participants with BMI of < 18.5 kg/m^2^ were considered underweight, between 18.5–24.9 kg/m^2^ as normal weight, BMI of 25–29.9 kg/m^2^ as overweight, and BMI of ≥ 30 kg/m^2^ as obese.

The PSG apparatus recorded the following indices: sleep efficiency (ratio of the total time spent asleep in a night compared with the total amount of time spent in bed), obstructive apnea (shows the number of obstructive apnea events per hour of sleep), central apnea index (shows the number of central apnea events per hour of sleep), hypopnea index and apnea and hypopnea index (AHI, average number of apneas and hypopneas per hour of sleep), respiratory effort-related arousal (RERA, reduction in airflow with resultant arousal but not meeting the desaturation criteria for hypopnea or apnea), respiratory disturbance index (RDI, average number of respiratory disturbances) (i.e., obstructive apneas, hypopneas, and RERAs) per hour of sleep, and periodic limb movements with arousal index. A registered sleep technician performed the polysomnographic studies, and scoring was made according to the American Academy of Sleep Medicine.^14^

### Statistical analysis

To describe the study results, qualitative variables are presented as frequency (percentage) and quantitative variables as mean ±  standard deviation (SD). To compare the frequency of categorical variables between the groups, the chi-square test and Fisher's exact test were used. The normal distribution of data was tested using the one-sample Kolmogorov–Smirnov test and equality of variances by Levene's test. Independent samples *t*-test was used to compare numerical variables. The linear regression model was used to examine the effect of variables on the study outcomes. The chi-square test results were used to evaluate the effect of variables on AHI, as studies have shown that the significance level of some variables may change in the multivariate analysis.^15–18^ We used multivariate analysis when a p-value of < 0.2 was observed in the univariate analysis. This analysis helps ensure that all pertinent and potentially predictive variables are evaluated. Then, backward analysis was used. The statistical software IBM SPSS Statistics for Windows version 21.0 (IBM Corp, Armonk, NY, USA) was used for the statistical analysis. P values of 0.05 or less were considered significant.

## Results

A total of 126 patients were included in the study, with a mean ±  SD age of 52.1 ± 12.73 (range, 15–87) years, of which 35.7% (N = 45) were female and 64.3% (N = 81) were male. The mean ±  SD age of the female and male participants was 52.93 ± 13.33 and 51.64 ± 12.53 years, respectively. A total of 96 (76.2%) participants had an AHI of ≥ 15/h, and 30 participants (23.8%) had an AHI of < 15/h; 100 (79.4%) patients had an RDI of ≥ 15/h, and 26 (20.6%) patients had an RDI < 15/h. The AHI was 44.13 ± 30.68, and the RDI was 47.34 ± 30.02.

AHI and RDI were not different based on patients’ sex (p>0.05). However, the mean age of the patients with AHI and RDI of ≥ 15/h were significantly higher than that of patients with AHI and RDI ≤ 15/h (p < 0.05), and the mean age was higher in those with severe disease (p = 0.039). The frequency of RDI and AHI severities were different between patients’ BMI categories, and most patients with AHI and RDI of ≥ 15/h had BMI of ≥ 30 kg/m^2^ (both p < 0.001).

Apnea was absent in 11 patients (8.7%) with a mean AHI of 2.46, mild in 10 (7.9%) patients with a mean AHI of 6.75, moderate in 9 (7.1%) patients with a mean AHI of 14.2, severe in 16 (12.7%) patients with a mean AHI of 22.76, and intense in 80 (63.5%) patients with a mean AHI of 62.03.

Of the 100 patients with RDI of ≥ 15/h, 95% had snoring, 97% had trouble sleeping, 83% had not used sedatives, 65% had nocturia, 65% had not experienced nightmares, 80% had observed apnea, 77% had awakened with a feeling of choking, 47% had restless legs, and 80% had daytime sleepiness. Of the 96 patients with AHI ≥ 15/h, 94.7% had snoring, 96.8% had sleep disorders, 92.9% had not used sedatives, 64.5% had nocturia, 63.5% has not experienced nightmares, 79.1% had observed apnea, 76% had awakened with a feeling of choking, 46.8% had restless legs, and 79.1% had daytime sleepiness. A significant association was found between the frequency of RDI and AHI < 15 or ≥ 15/h and snoring, nocturia, observed apnea, substance abuse, cardiac disease, and hypertension.

The frequency of RDI and AHI severities were different between patients with and without snoring (p = 0.017 and 0.028), sleep disorder (p = 0.033 and 0.011), and nocturia (p = 0.001 and 0.011), but no difference in frequency was found regarding the presence or absence of nightmares (p = 0.87 and 0.073), observed apnea (p = 0.08 and 0.18), awakening with a feeling of choking (p = 0.56 and 0.32), restless legs (p = 0.93 and 0.97), and daytime sleepiness (p = 0.28 and 0.23), respectively; (data not shown). RDI severity was not significantly different based on sedatives (p = 0.079), but the frequency of AHI severities was different based on the use of sedatives (p = 0.039). The history of the sedatives is unknown.

Of the 19 patients who smoked, 63.1% had RDI of ≥ 15/h and 57.8% AHI of ≥ 15/h. Of the 100 patients with RDI of ≥ 15/h, 97% had no substance abuse, and of the 96 patients with AHI of ≥ 15/h, 96.8% had no substance abuse. The frequency of AHI severities was different between patients with and without smoking (p = 0.039), but that of RDI was not different (p = 0.063) (Table 1). The frequency of RDI and AHI severities were different between patients with and without substance abuse (both p = 0.01) (Table 2).

Among several underlying diseases, the frequency of AHI and RDI of < 15 or ≥ 15/h was only different based on hypertension and cardiac disease. Of the 61 patients with hypertension, 91.8% had an RDI of ≥ 15/h, and 88.5% had an AHI of ≥ 15/h. Of the 37 patients with cardiac disease, 91.8% had RDI of ≥ 15/h, and 89.2% had an AHI of ≥ 15/h (p < 0.05). Among the underlying diseases, the frequency of severities of RDI and AHI were only different based on hypertension (p = 0.004 and 0.02, respectively).

The frequency of obstructive, central, and hypopnea scores of < 15 or ≥ 15/h according to RDI and AHI of < 15 or ≥ 15/h shows a significant difference with respect to obstructive and hypopnea factors (p < 0.05), but not to the central factor (p>0.05). The frequency of the severities of obstructive and hypopneas was significantly different based on RDI and AHI of < 15 or ≥ 15/h (p < 0.001), while the central factor was not (p = 0.7).

The results of the univariate regression analysis showed snoring (p = 0.012), nocturia (p = 0.001), observed apnea (p = 0.035), smoking (p = 0.045), substance abuse (p = 0.034), hypertension (p = 0.002), and BMI (p < 0.001) as influential factors on AHI of ≥ 15/h, and the multivariate regression analysis showed nocturia (p = 0.045) and BMI (p = 0.006) as significant factors. In the univariate analysis, a significant association was found between the mean RERA index and awakening with choking (p = 0.039), nocturia (p = 0.009), daytime sleepiness (p = 0.046), substance abuse (p = 0.023), and diabetes mellitus (p = 0.027), but they were not significant in the multivariate analysis. Finally, the enter method showed that only reflux was the factor that affected the RERA index with the following model: RERA = − 2.09+3.09 ×  reflux.

Testing variables with p>0.2, including sex, sleep disorder, nightmare, awakening with choking, cardiac disease, lung disease, stroke, depression, and age, showed no significant association with AHI (p>0.05; data not shown). The association between RDI and AHI, and smoking and substance abuse were shown in tables 3 and 4.

The association of AHI with variables with p < 0.2 is shown in Table 5. As demonstrated, substance abuse and BMI were associated with AHI by the following model: AHI = − 9.54 – 24.49 ×  substance abuse +1.17 ×  BMI.

## Discussion

Of the 126 patients undergoing PSG, 76.2% had an AHI of ≥ 15/h and 79.4% an RDI of ≥ 15/h. Meanwhile, apnea was severe in 12.7% and intense in 63.5%, which shows that the inclusion of patients based on the parameters mentioned in the Methods can successfully estimate the patients who are at a high risk of severe and intense obstructive sleep apneas. In this study, several risk factors have been evaluated and compared between patients with AHI and RDI of < 15 or ≥ 15/h. The results showed that sex was not a compelling factor in obstructive sleep apnea severity, while RDI and AHI increased with advancing age. Previous studies have suggested a higher prevalence and severity of obstructive sleep apnea in men than in women, which was supposed to underlie the sex differences in the upper airway anatomy, body fat distribution, and hormones.^19,20^ A study also suggested that women are more symptomatic at a lower stage of obstructive sleep apnea and are thus diagnosed before the disease becomes very severe.^21^ Nevertheless, some authors suggested that this sex difference was observable only in patients with AHI 5–15/h,^19^ which can justify the lack of difference found based on patients’ sex, as shown in the present study. The higher severity of obstructive sleep apnea in older patients, as suggested in the present study, has been confirmed in previous studies and was associated with worse cardiocerebrovascular, cognitive, and functional outcomes.^22,23^ Deng et al. suggested the association of higher age with obstructive sleep apnea exacerbation in different age categories in men ( ≤ 40 years) and women (aged 45–53 years).^24^

In this study, BMI was identified as an essential factor associated with the severity of obstructive sleep apnea. Not only did the AHI and RDI increase by the rise in patients’ BMI, but the results of the multiple regression analysis have also determined BMI as an essential predictor of AHI of ≥ 15/h. Obesity is one of the well-recognized risk factors of obstructive sleep apnea. Patients with a higher BMI are at increased risk of obstructive sleep apne,^25^ and weight loss is considered one of the cornerstones of the treatment strategies of obstructive sleep apnea.^26^

In this study, several sleep-related problems have been observed with a higher frequency in patients with AHI and RDI of ≥ 15/h, including snoring, nocturia, observed apnea, awakening with a feeling of choking, and restless legs during sleep. These results emphasize including these factors in the questionnaire used for the clinical estimation of obstructive sleep apnea severity and the need for PSG. In this study, snoring was observed in 97% of the patients with AHI of ≥ 15/h and RDI of ≥ 15/h. Romero et al. suggested that snoring can predict obstructive sleep apnea with a sensitivity of 82.6% and specificity of 43%, which confirm the importance of snoring as a predictive factor of obstructive sleep apnea,^27^ as also proposed in the present study. These authors also suggested that nocturia can predict obstructive sleep apnea with a sensitivity of 84.8% and specificity of 22.4%,^27^ which confirms our observation of nocturia in 65% of the patients with AHI of ≥ 15/h and RDI of ≥ 15/h. A study suggested that continuous positive airway pressure can be an effective treatment for nocturia in patients with obstructive sleep apnea,^28^ which, in line with the results of the present study, emphasizes the inclusion of nocturia in the clinical screening of patients with obstructive sleep apnea. In this study, the results of the multiple regression analysis showed nocturia as a significant and independent predictor of obstructive sleep apnea. This finding was also reported by Romero et al. who suggested nocturia frequency as a predictor of AHI score and obstructive sleep apnea severity, independent of other factors, such as sex, age, BMI, and snoring,^27^ which is consistent with the results of the present study and emphasizes the association of nocturia with obstructive sleep apnea severity. According to the evidence from an animal investigation, the oxidative stress of the bladder is the contributing factor for the association of nocturia and obstructive sleep apnea.^27^ Moreover, further studies are required to determine the pathophysiology of the association between nocturia and obstructive sleep apnea.

Awakening with a feeling of choking, observed in 76% of the patients with AHI of ≥ 15/h and 77% of those with RDI of ≥ 15/h, was another important factor in the present study and considered one of the clinical signs of obstructive sleep apnea severity that should be considered in the clinical assessment of obstructive sleep apnea.^29^ Awakening with a feeling of choking is related to the pathophysiology of obstructive sleep apnea, i.e., airway obstruction during sleep, which results in inadequate alveolar ventilation, reduced oxygen saturation, and partial increase in carbon dioxide (CO_2_) that terminate with the patients’ arousal and cause the feeling of choking for the patient.^30^ These factors also cause daytime sleepiness, lack of concentration, tiredness, and unrefreshed feeling at daytime.^31^ In the present study, 80% of the patients with AHI and RDI of ≥ 15/h reported daytime sleepiness, which emphasizes the effect of obstructive sleep apnea on the social function of the individual and refers to the necessity of on-time diagnosis and treatment. Restless legs during sleep was another factor frequently observed in patients with AHI and RDI of ≥ 15/h in our study, which is consistent with the results of previous studies that report a high frequency of restless leg syndrome (RLS) and periodic leg movements during sleep in patients with obstructive sleep apnea. However, the exact mechanism of this association is still unclear.^31,32^ A study reported that the treatment of obstructive sleep apnea with upper airway stimulation can help resolve RLS in these patients, which refers to the association of RLS with obstructive sleep apnea.^33^ Moreover, further studies should understand the underlying pathophysiology of this association. Most of the abovementioned factors are reported by the patients, while apnea observed by others, usually family members, can be an essential factor in the early diagnosis of obstructive sleep apnea.^34^ However, the patient should sleep beside a companion, and it might not be reportable in patients who sleep alone. In the meantime, a review of studies determined observed apnea as one of the four crucial factors that increase the methodological value for studies that have evaluated obstructive sleep apnea.^35^

Considering the association of obstructive sleep apnea with medical diseases, our study showed cardiac diseases and hypertension as the two crucial diseases associated with the severity of obstructive sleep apnea. According to available evidence, a mutual relationship exists between hypertension and obstructive sleep apnea. Hypertension is one of the risk factors of obstructive sleep apnea. OSA is also a risk factor for hypertension, which has been attributed to excess aldosterone, resulting in the accumulation of fluid within the neck and increased upper airway resistance in patients with obstructive sleep apnea.^36^ Furthermore, there is strong evidence on the effect of obstructive sleep apnea on the progression of atherosclerosis,^5^ coronary events, and cardiovascular death,^6,7^ which confirm the results of the present study regarding the higher frequency of cardiac diseases in patients with high AHI and RDI.

An interesting finding in this study was the lower frequency of nightmares in patients with higher AHI and RDI. Pagel et al. have also reported this finding. It was attributed to the cognitive impairment in these patients, which impaired their nightmare recall and the suppressed REM in patients with obstructive sleep apnea.^37^

### Limitations

As limitations, this study had a small sample size and retrospectively evaluated the variables, which limited the suggestion of causal correlations between the study variables. In addition, patients were not randomly included in the study. All patients were undergoing PSG at one center, limiting the generalizability of the study results. Finally, follow-up was not considered.

## Conclusion

OSAS is associated with several systemic comorbidities, such as obesity, hypertension, and cardiac diseases, which must be considered in the treatment strategies of each patient. Furthermore, this study showed some factors associated with severe obstructive sleep apnea that must be included in the clinical assessment of the severity of obstructive sleep apnea in suspected patients; these factors included snoring, nocturia, awakening with a feeling of choking, and observed apnea. Patients positive for these factors are strongly recommended to undergo PSG. Further studies are needed to reveal how sedative use, smoking, and substance abuse could change the severity of OSAS.

### Declarations

### Acknowledgments

The present article was extracted from the thesis written by Soroush Bagheri.

### Conflicting Interest

The authors declare that they have no conflict of interest.

### Funding/Support

The Vice Chancellor for Shiraz University of Medical Sciences financially supported this study (Grant No. 16702).

## Figures and Tables

**Table 1:** Association between RDI and AHI and smoking

Factor	Apnea	Smoking		Total	p-value

		Non-smoker (n = 107)	Smoker (n = 19)		

RDI, No. (%)	Not	4(3.2)	0(0)	4(3.2)	

	Mild	6(4.8)	3(2.4)	9(7.1)	

	Moderate	9(7.1)	4(3.2)	13(10.3)	0.063

	Severe	14(11.1)	4(3.2)	18(14.3)	

	Very severe	74(58.7)	8(6.3)	82(65.1)	

AHI, No. (%)	Not	10(7.9)	1(0.8)	11(8.7)	

	Mild	6(4.8)	4(3.2)	10(7.9)	

	Moderate	6(4.8)	3(2.4)	9(7.1)	0.039

	Severe	13(10.3)	3(2.4)	16(12.7)	

	Very severe	72(57.1)	8(6.3)	80(63.5)	


RDI, respiratory disturbance index; AHI, apnea–hypopnea index.

**Table 2:** Association between RDI and AHI and substance abuse

Factor	Apnea	Substance abuse		Total	p-value

		No (n = 119)	Yes (n = 7)		

RDI, No. (%)	Not	4(3.2)	0(0)	4(3.2)	

	Mild	7(5.6)	2(1.6)	9(7.1)	

	Moderate	11(8.7)	2(1.6)	13(10.3)	0.01

	Severe	16(12.7)	2(1.6)	18(14.3)	

	Very severe	81(64.3)	1(0.8)	82(65.1)	

AHI, No. (%)	Not	10(7.9)	1(0.8)	11(8.7)	

	Mild	9(7.1)	1(0.8)	10(7.9)	

	Moderate	7(5.6)	2(1.6)	9(7.1)	0.01

	Severe	14(11.1)	2(1.6)	16(12.7)	

	Very severe	79(62.7)	1(0.8)	80(63.5)	


RDI, respiratory disturbance index; AHI, apnea–hypopnea index.

